# Molecular analysis of antigen presentation machinery in circulating tumor cells from renal cell carcinoma and prostate cancer

**DOI:** 10.1186/2051-1426-1-S1-P57

**Published:** 2013-11-07

**Authors:** Joshua M Lang, Jacob T Tokar, Jamie Sperger, Benjamin P Casavant, Scott M Berry, Lindsay N Strotman, David J Beebe

**Affiliations:** 1 Department of Medicine, University of Wisconsin, Madison, WI, USA; 2 Biomedical Engineering, University of Wisconsin, Madison, WI, USA; 3 Carbone Cancer Center, University of Wisconsin, Madison, WI, USA

## 

Aberrant expression of antigen presentation machinery (APM) has been closely associated with more aggressive tumors and may inhibit the therapeutic response to T-cell based immunotherapies. Furthermore, the selective pressure of immunotherapies may select for resistant clones that down regulate expression of APM as means of therapeutic resistance. This report characterizes a microfluidic assay for the screening and serial assessment of APM in circulating tumor cells (CTCs) from patients with advanced renal cell carcinoma and prostate cancer. Cell surface expression of MHC class I is identified in both renal cell and prostate cancer CTCs. Gene expression analysis for components of APM in CTCs is also validated using quantitative RT-PCR. The results of these studies suggest a unique CTC biomarker that may serve as a predictive and pharmacodynamic biomarker for use in clinical trials with T-cell based immunotherapies. Future studies will focus on the development of assays for markers of resistance to checkpoint inhibitors and expression of tumor associated antigens.

